# Supplementary material for: Detection of the Cyclic Imines Pinnatoxin G, 13-Desmethyl Spirolide C and 20-Methyl Spirolide G in Bivalve Molluscs from Great Britain
Source: Mar Drugs. 2024 Dec 12;22(12):556. doi: 10.3390/md22120556 (PMC11677300; doi:10.3390/md22120556)
Supplement: Supplementary file 1 [file marinedrugs-22-00556-s001.zip › marinedrugs-3315805-supplementary.pdf]

Supplementary materials

PnTx G vs month

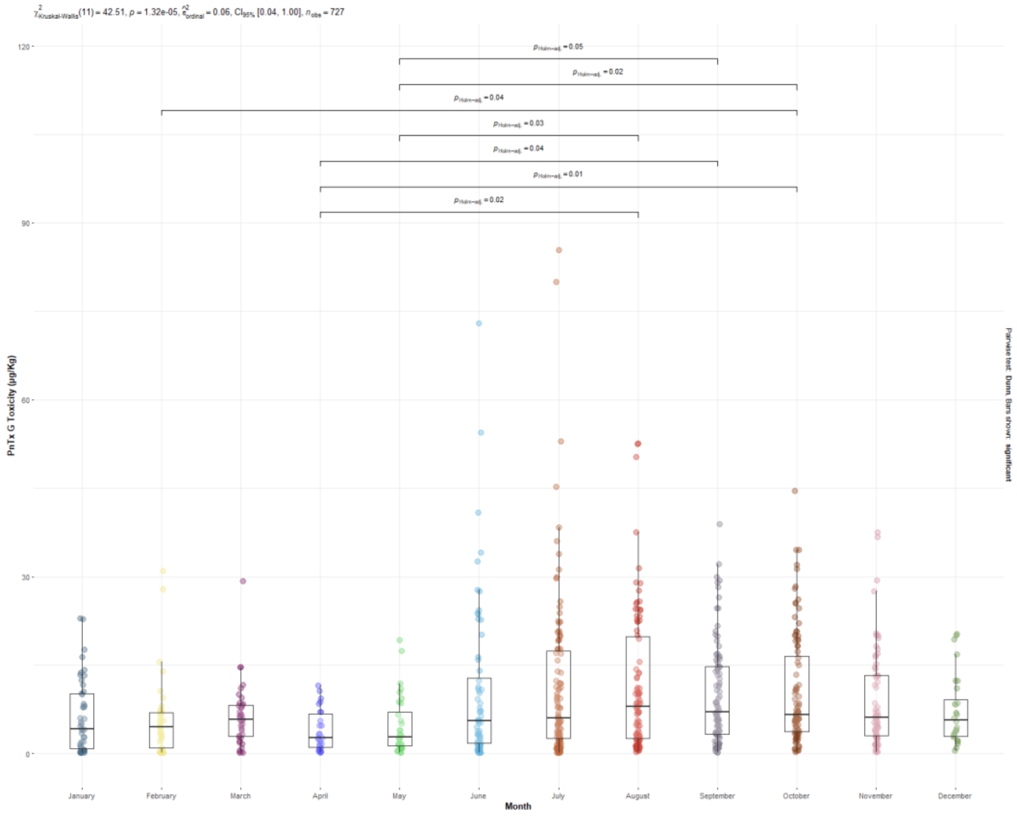

Figure S1: Kruskal-Wallis with Dunn pairwise test output when comparing months and PnTx G toxicity.

From Figure S1 we can see that $P < 0.01$ so we accept the alternate hypothesis that there is a strong statistically significant difference between month and PnTx G concentration with a 99% confidence level. There are 7 statistically significant differences in concentrations when comparing between months. Interpretation of the epsilon squared value showed that month had a medium effect size on PnTx G toxicity.

SPX 1 vs month

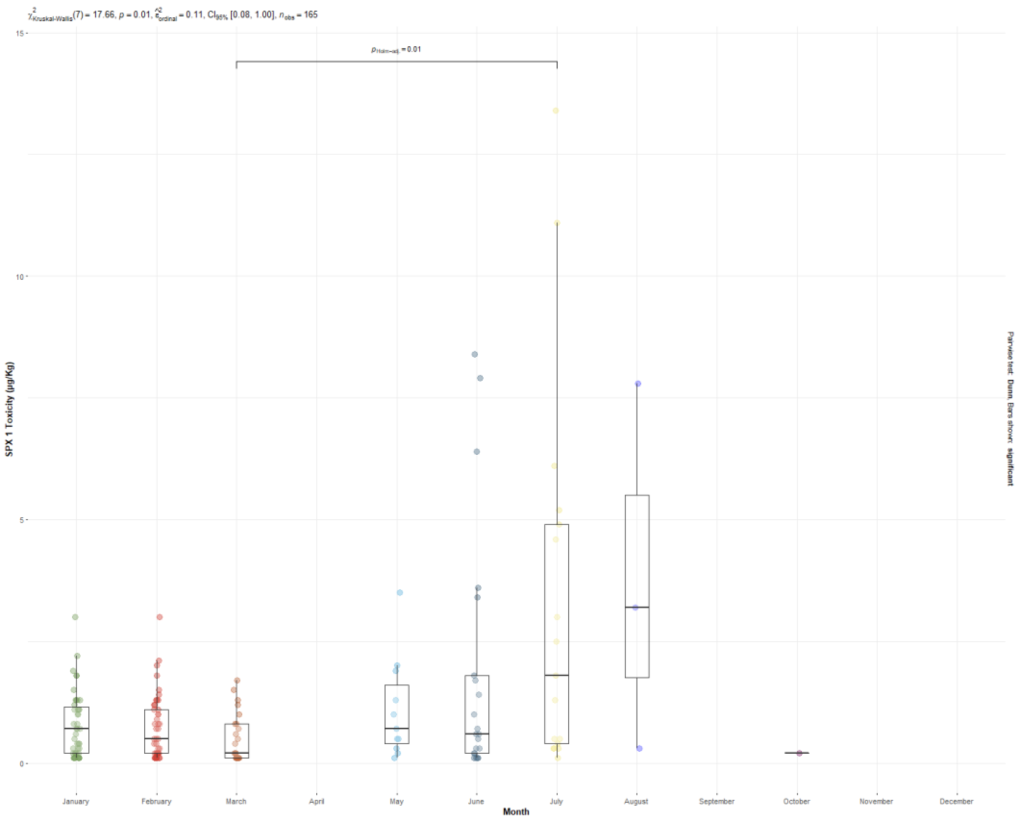

Figure S2: Kruskal-Wallis with Dunn pairwise test output when comparing months and SPX 1 toxicity.

From Figure S2 we can see that P = 0.01 so we accept the alternate hypothesis that there is a statistically significant difference between month and SPX 1 concentration with a 99% confidence level. There is 1 statistically significant difference in concentrations when comparing between months: July and March. Interpretation of the epsilon squared value showed that month had a medium effect on SPX 1 toxicity.

20-methyl spirolide G vs month

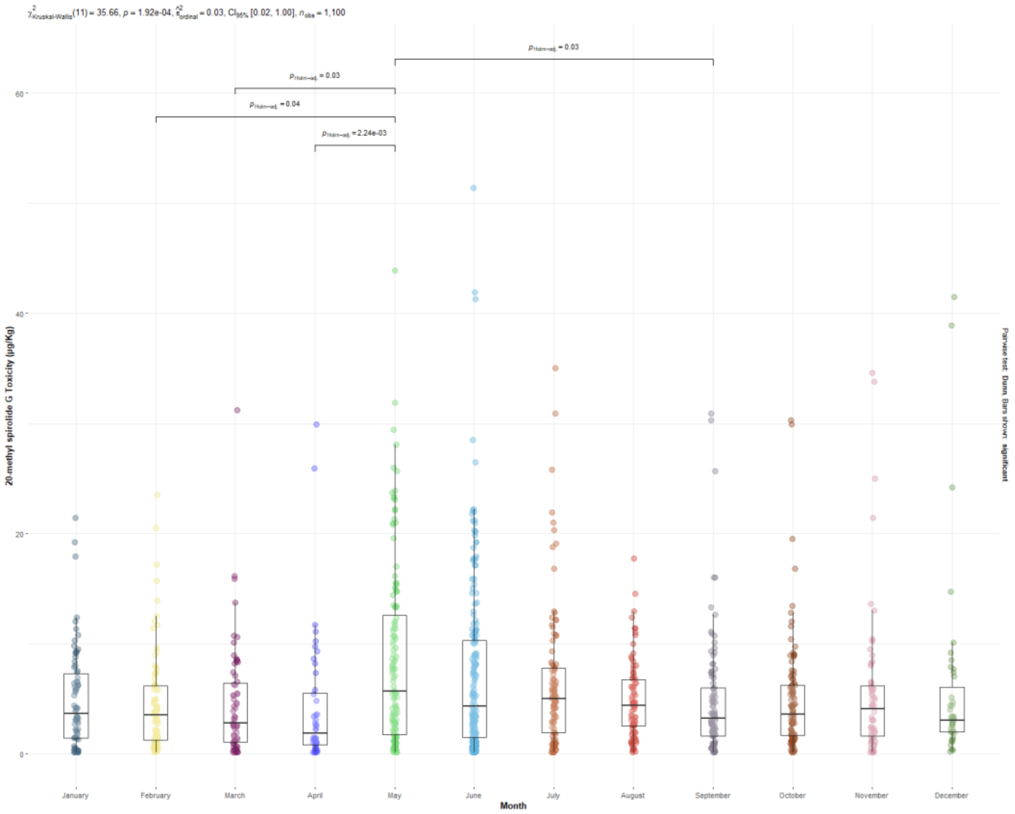

Figure S3: Kruskal-Wallis with Dunn pairwise test output when comparing months and 20-methyl spirolide G toxicity.

From Figure S3 we can see that P < 0.01 so we accept the alternate hypothesis that there is a strong statistically significant difference between month and 20-Me-SPX G concentration with a 99% confidence level. There are 4 statistically significant differences in concentrations when comparing between months. Interpretation of the epsilon squared value showed that month had a small effect on 20-MeSPX G toxicity.

PnTx G vs season

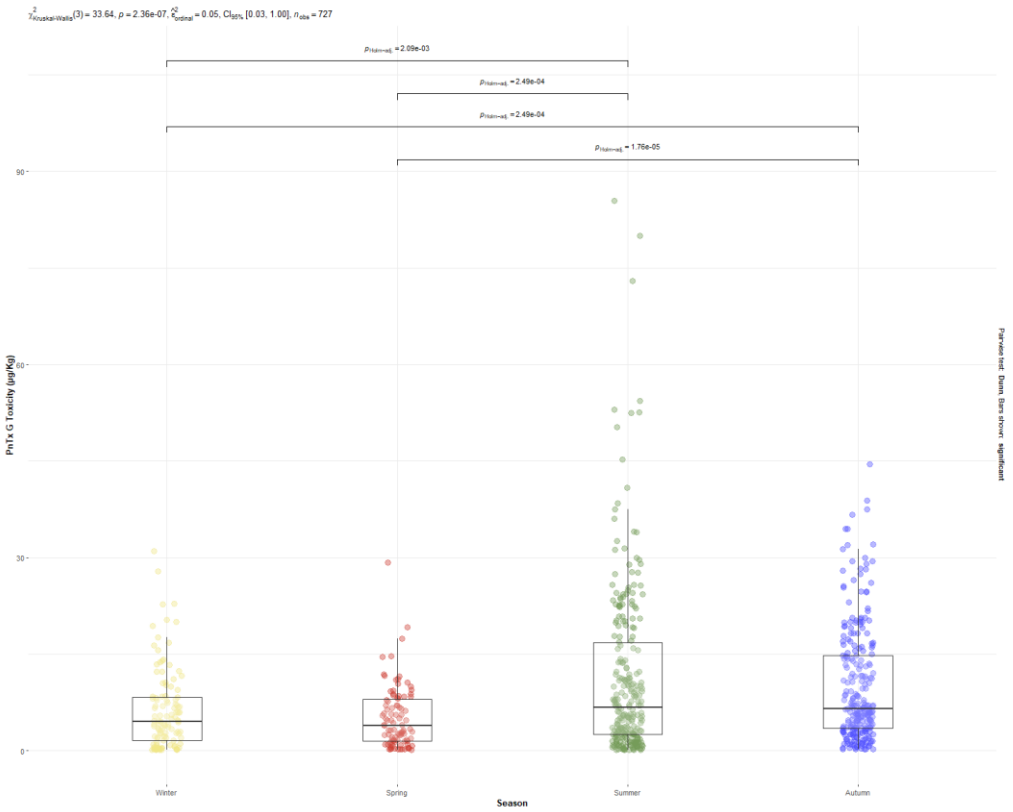

Figure S4: Kruskal-Wallis with Dunn pairwise test output when comparing season and PnTx G toxicity.

From Figure S4 we can see that P < 0.01 so we accept the alternate hypothesis that there is a strong statistically significant difference between season and PnTx G concentration with a 99% confidence level. There are 4 statistically significant differences in concentrations when comparing between seasons. Interpretation of the epsilon squared value showed that season had a small effect on PnTx G toxicity

SPX 1 vs season

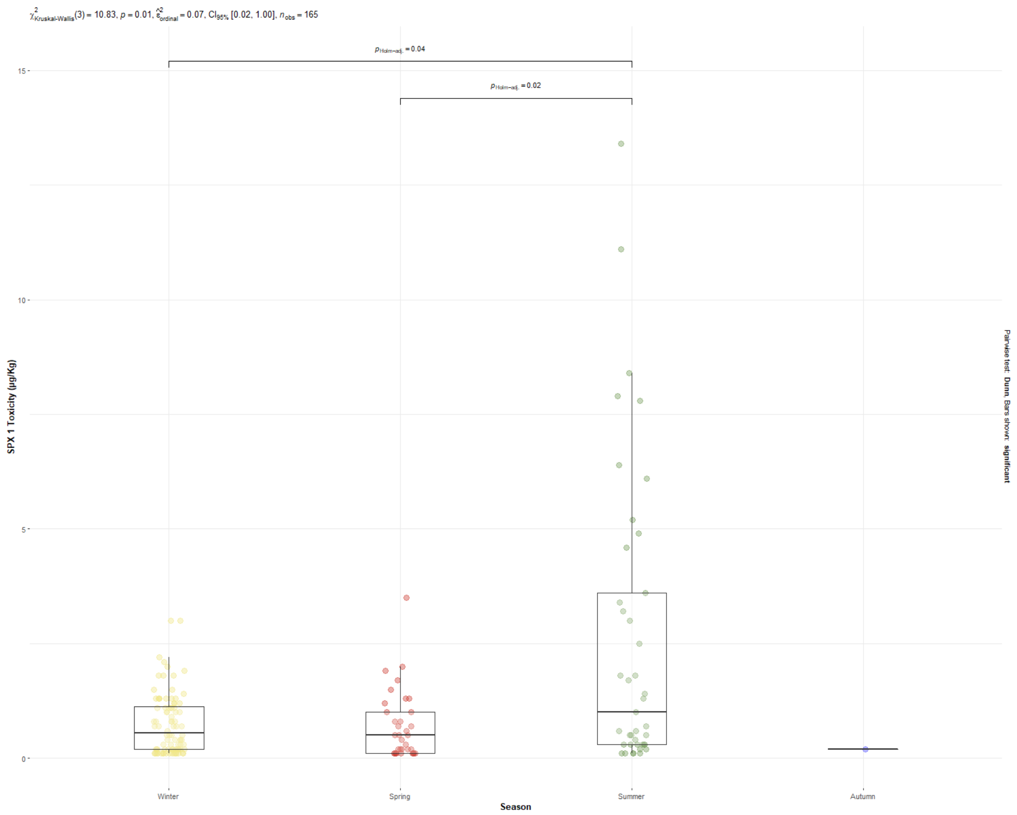

Figure S5: Kruskal-Wallis with Dunn pairwise test output when comparing season and SPX 1 toxicity.

From Figure S5 we can see that P = 0.01 so we accept the alternate hypothesis that there is a statistically significant difference between season and SPX 1 concentration with a 99% confidence level. There are 2 statistically significant differences in concentrations when comparing between seasons: Summer and winter; and summer and spring. Interpretation of the epsilon squared value showed that season had a medium effect on SPX 1 toxicity.

20-methyl spirolide G vs season

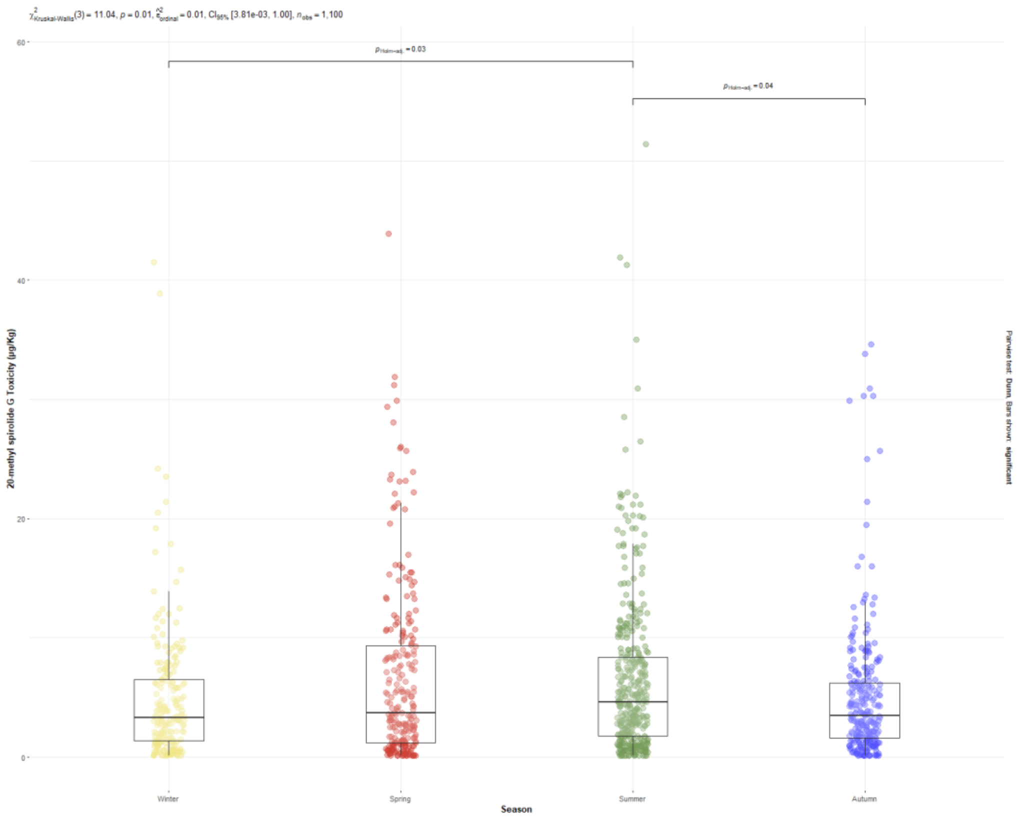

Figure S6: Kruskal-Wallis with Dunn pairwise test output when comparing season and 20-methyl spirolide G toxicity.

From Figure S6 we can see that P = 0.01 so we accept the alternate hypothesis that there is a statistically significant difference between seasons and 20-Me-SPX G concentration with a 99% confidence level. There are 2 statistically significant differences in concentrations when comparing between seasons: Summer and winter; and summer and autumn. Interpretation of the epsilon squared value showed that season had a small effect on 20-Me-SPX G toxicity.

PnTx G vs species

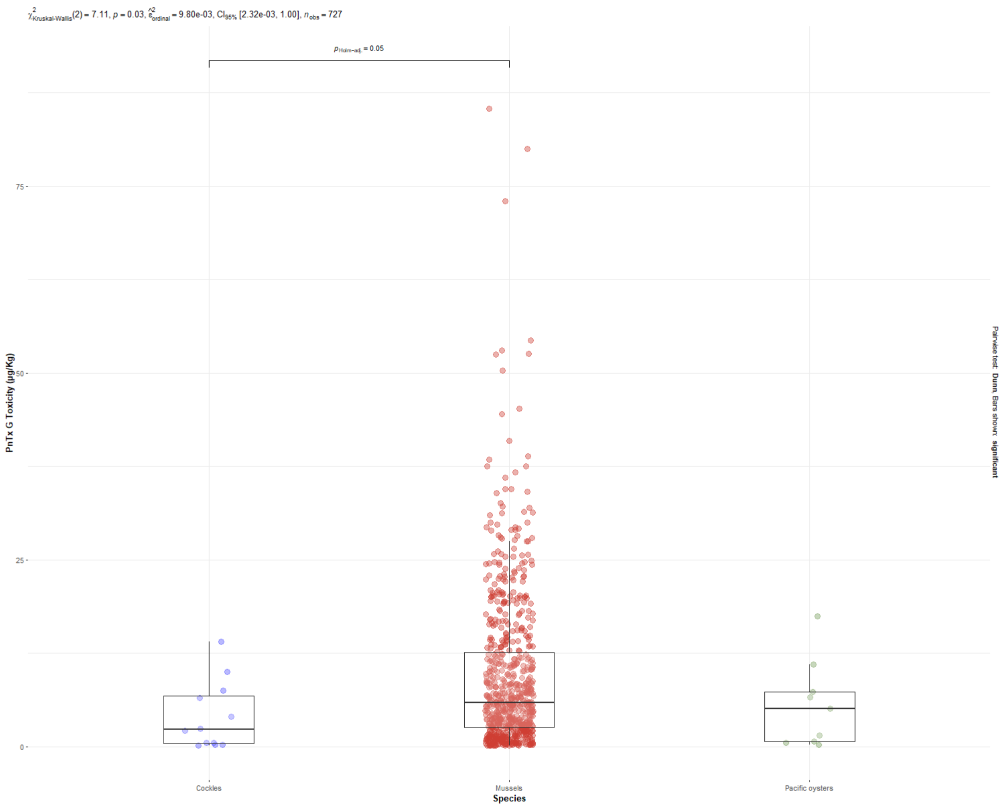

Figure S7: Kruskal-Wallis with Dunn pairwise test output when comparing species and PnTx G toxicity.

From Figure S7 we can see that P < 0.05 so we accept the alternate hypothesis that there is a statistically significant difference between species and PnTx G concentration with a 95% confidence level. There is 1 statistically significant difference in concentrations when comparing between species; mussels and cockles. Interpretation of the epsilon squared value showed that species had a very small effect on PnTx G toxicity.

SPX 1 vs species

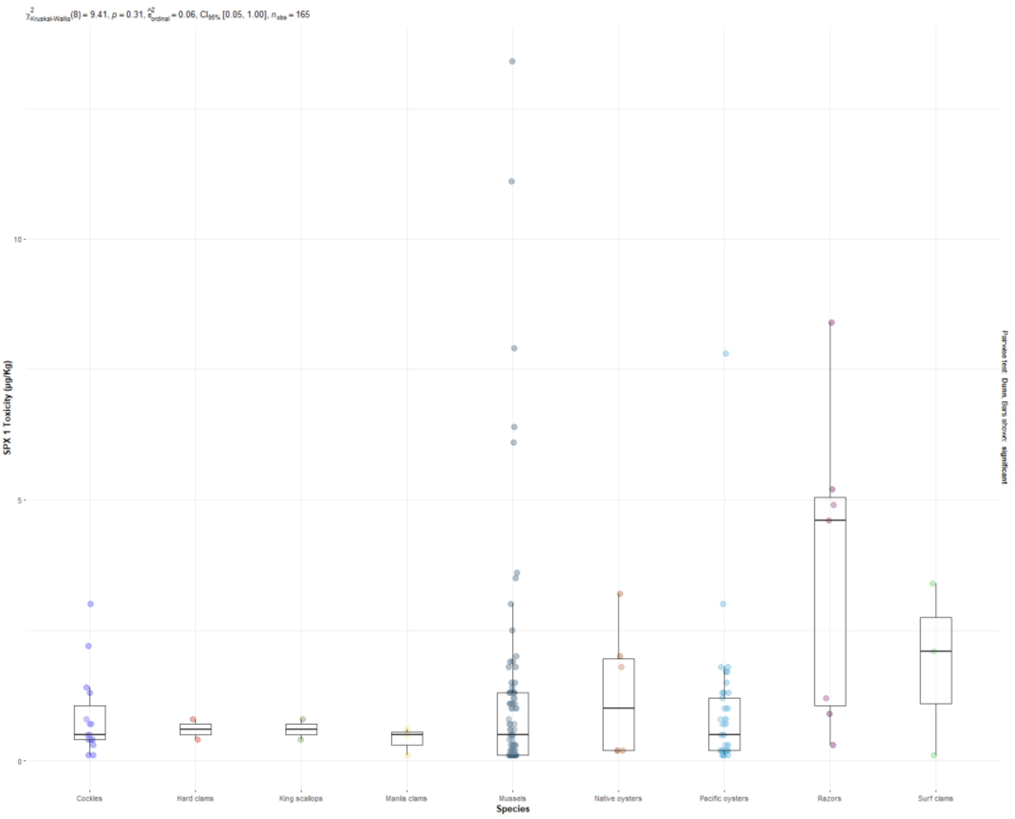

Figure S8: Kruskal-Wallis with Dunn pairwise test output when comparing species and SPX 1 toxicity.

From Figure S8 we can see that P > 0.05 so we accept the null hypothesis that there is no statistically significant difference between species and SPX 1 concentration with no statistically significant differences in concentrations when comparing between species. Interpretation of the epsilon squared value showed that species had a medium effect on SPX 1 toxicity.

20-methyl spirolide G vs species

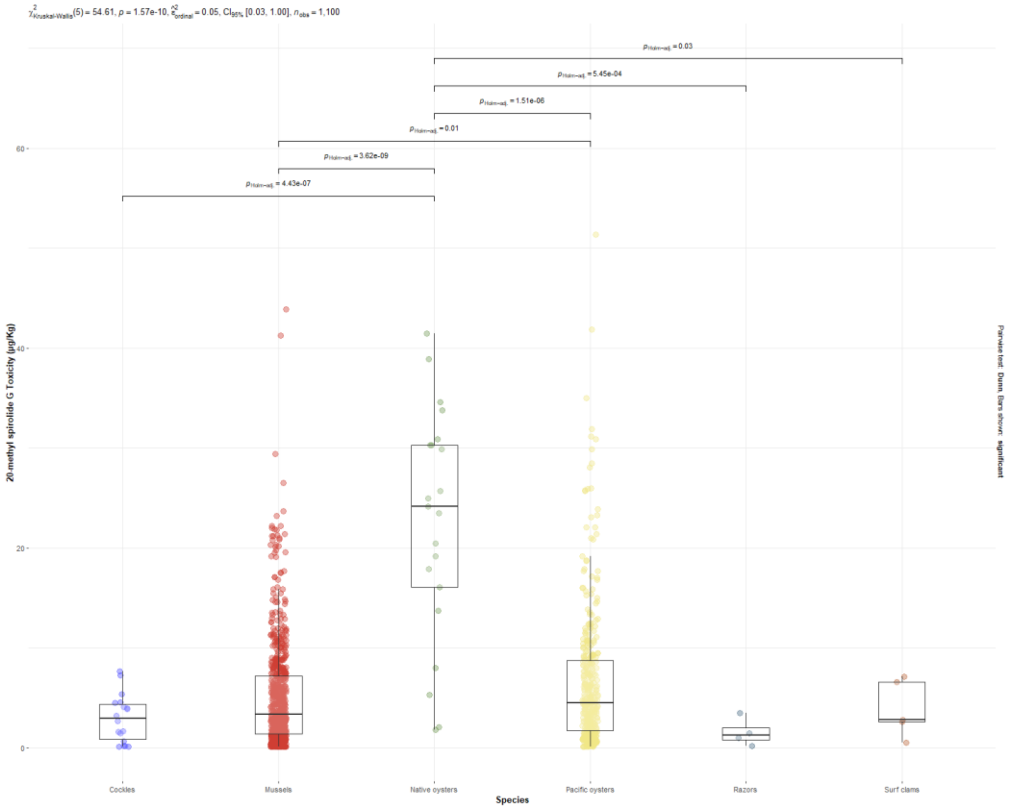

Figure S9: Kruskal-Wallis with Dunn pairwise test output when comparing species and 20-methyl spirolide G toxicity.

From Figure S9 we can see that P < 0.01 so we accept the alternate hypothesis that there is a strong statistically significant difference between species and 20-Me-SPX G concentration with a 99% confidence level. There are 6 statistically significant differences in concentrations when comparing between species. Interpretation of the epsilon squared value showed that species had a small effect on 20-Me-SPX G toxicity.

Temporal analysis – seasons

Table S1: Comparison of temporal (seasonal) toxin concentrations in 2018.

		Seasons			
		**Winter**	**Spring**	**Summer**	**Autumn**
Total Sampled each season		411	574	886	800
PnTxG (µg/L)	Mean	6.20	5.12	11.34	9.98
	SD	6.23	4.87	13.37	8.90
	Count	117.00	96.00	262.00	254.00
	% pos (month)	28.47%	16.72%	29.57%	31.75%
SPX1 (µg/L)	Mean	0.67	0.67	2.55	0.20
	SD	0.67	0.75	3.28	0.00
	Count	99.00	36.00	42.00	1.00
	% pos (month)	24.09%	6.27%	4.74%	0.13%
20-Me-SPX-G (µg/L)	Mean	4.93	6.63	6.48	5.07
	SD	5.69	7.45	6.83	5.75
	Count	209.00	239.00	388.00	265.00
	% pos (month)	50.85%	41.64%	43.79%	33.13%

Winter = January, February, December
Spring = March, April, May
Summer = June, July August
Autumn = September, October, November

Total Ion Chromatograms

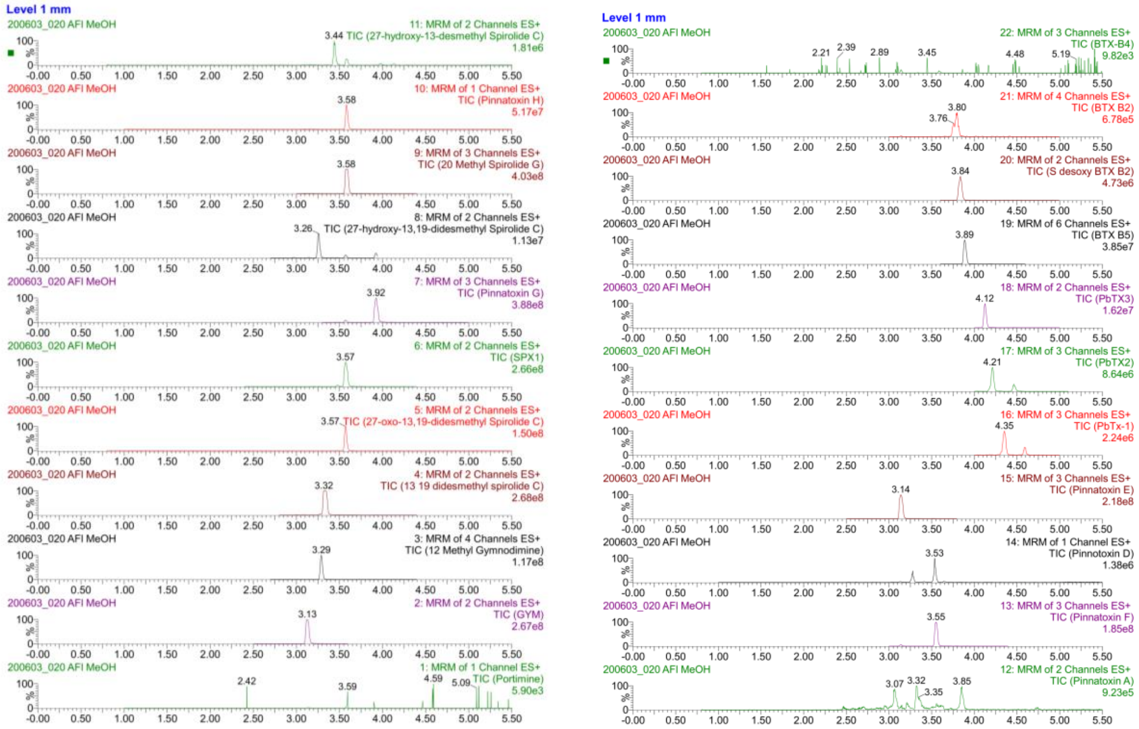

Figure S10: Total ion chromatogram for each analyte in the level 1 standards.

Geographic map and corresponding regions.

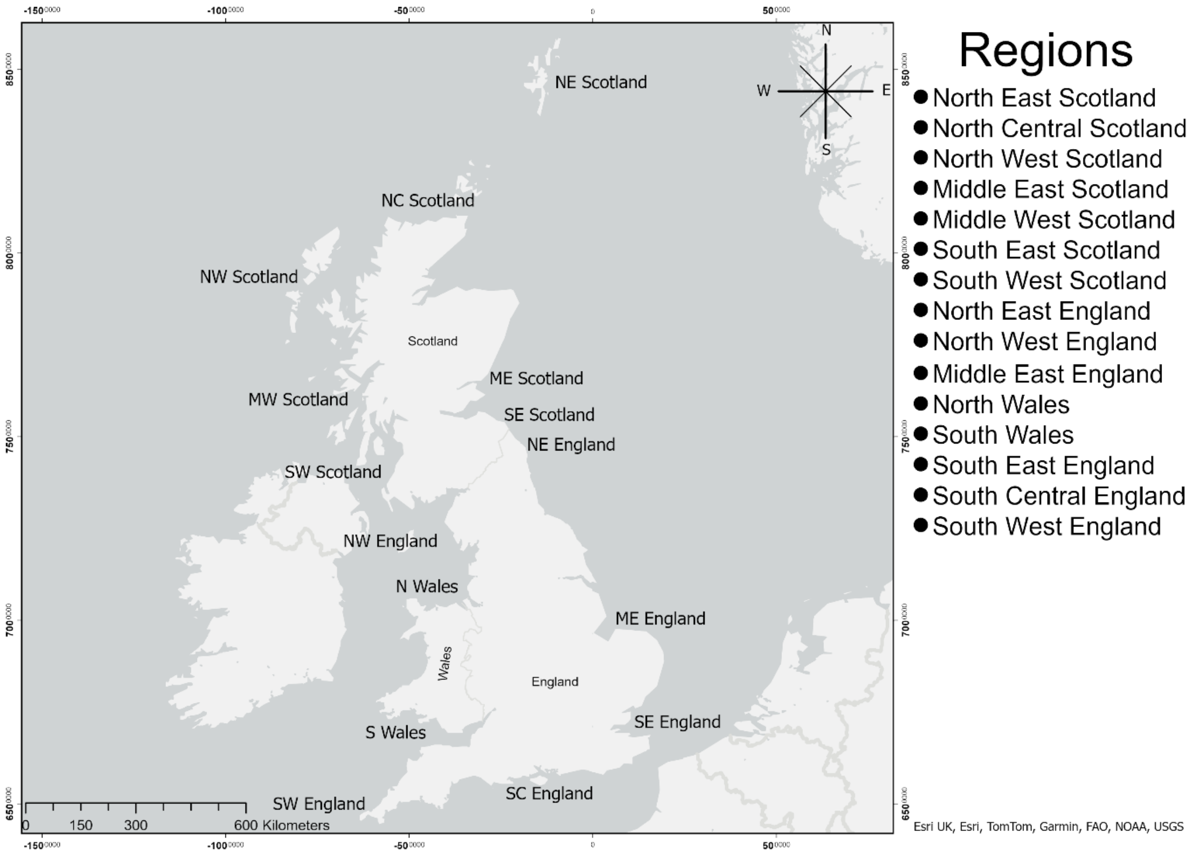

Figure S11: Map of the UK showing geographic regions as used in the current study.

PnTx G concentrations after hydrolyses

Table S2: Summary of PnTx G concentrations after hydrolyses.

Sample	Free PnTx G (µg/kg)	Total PnTx G (µg /kg)	Difference
1	17.6	19.3	8.6%
2	18.1	20.0	9.5%
3	18.8	26.5	29.1%
4	19.4	19.9	2.4%
5	19.4	20.5	5.4%
6	20.6	21.9	5.8%
7	21.2	21.8	2.5%
8	22.2	27.9	20.4%
9	22.5	23.0	2.2%
10	23.1	23.9	3.2%
11	25.2	25.6	1.7%
12	25.8	30.0	14.0%
13	26.4	28.6	7.8%
14	26.6	29.5	9.8%
15	28.3	29.8	4.9%
16	29.2	32.3	9.5%
17	30.7	32.8	6.3%
18	32.4	38.4	15.6%
19	34.3	37.5	8.5%
20	35.7	39.8	10.2%
21	38.2	39.9	4.2%
Mean	25.5	28.0	8.6%
